# MicroRNA-466c-3p exerts protective effect on neuronal apoptosis and improves functional recovery post spinal cord injury via mitochondrial apoptotic pathway

**DOI:** 10.1186/s13568-020-01033-3

**Published:** 2020-06-15

**Authors:** Yan An, Jianing Li, Qiang Yuan, Mingxing Fan

**Affiliations:** Department of Spine Surgery, Beijing Jishuitan Hospital, Beijing, 100035 China

**Keywords:** miR-466c-3p, Spinal cord injury, Bcl-2, Apoptosis

## Abstract

Spinal cord injury (SCI) is involved with abnormal expression of miRNAs (miRs) which are responsible for some II^ry^ injury responses which include apoptosis, inflammation and oxidative stress. Mechanisms involving miRs induced apoptosis still needs to be investigated. In the present work we developed a rat model of SCI, followed by microarray analysis for expression of miRs at various time points after SCI. The locomotor activity was assessed by Basso, Beattie and Bresnahan score, lesion volume was analyzed by cresyl violet staining and TUNEL staining for extent of apoptosis at various time points of post SCI. Numbers of miRs were altered after 2 weeks of SCI among which miR-466c-3p was the most significantly down-regulated. Transfection with miR-466c-3p mimics caused overexpression of miR-466c-3p, also improvement in functional recovery, decrease in apoptosis of neuronal cells and lesion size was observed in SCI rats. The Luciferase assay suggested that miR-466c-3p suppressed the expression of Bcl-2 (apoptosis regulator). It was also evidenced that upon restoring the levels of Bcl-2 with the help of pc-DNA3-Bcl-2 halted the attenuating action of miR-466c-3p in hydrogen peroxide exposed N9 microglia cells. The findings suggested that miR-466c-3p may inhibit mitochondrial apoptotic pathway via blocking Bcl-2 and cleaved capase-9/-3in rats after SCI. Altogether, the results suggested that miR-466c-3p may exert attenuating effect on functional recovery and inhibit the apoptosis of neuronal cells via halting the mitochondrial apoptosis cascade in SCI rats indicating that miR-466c-3p can be attractive therapeutic candidate in treating SCI.

## Introduction

Spinal cord injury (SCI) is a common injury of spinal cord leading to permanent disability which includes paralysis, loss of sensitivity, control and movements in the lower part of body below the affected region (Chen et al. 2016). The global rate of SCI is alarming; treatment for SCI is biggest challenge with the neurologists world-wide (Thuret et al. 2006). Apoptosis of neuronal cells is the major hurdle in SCI as the neurons play an important role in functional as well as physical deficits (Blight 2002; Rabchevsky et al. 2011), hence developing novel therapeutic approaches targeting and halting apoptosis is of outmost importance in managing SCI.

Apoptosis is a phenomenon in cells also called as programmed cell death; it is an important process which affects the neuronal tissue damage after SCI (Kawabata et al. 2010). However earlier it has been reported that both mitochondrial pathway and death receptor cause apoptosis (Li et al. 2000). Previously it has been evidenced that Bcl-2 members arbitrate apoptosis signals by involving anti–apoptosis proteins like B cell lymphoma and proteins such as Bax and Bcl-2 which are agonist of cell death in the mitochondrial cascade (Reuter et al. 2008). The degeneration of mitochondrial membrane potential is the main process of mitochondrial apoptotic pathway which leads to transfer of cytochrome-c to the cytosol from the mitochondria (Balaban et al. 2005). In addition to this, cytochrome-c along with deoxy ATP and apoptosis protease triggering factor-1 converts cleaved pro-caspase-9 into active-caspase-9 in the cytosol (Li et al. 2004). Further, this active-caspase-9 causes cleavage of caspase-3/-6/-7 (Nicholson and Thornberry 1997). Hence the release of cytochrome-c is an important step for the activation of pro-caspase-9 in apoptosis of cells.

miRNAs (miRs) are identified to be small noncoding RNAs having single strand measuring about 21–24 nucleotides. These micro-molecules are responsible for modulating the expression of target genes at post-transcriptional level by causing degradation of RNA or suppressing the translation (Croce 2009; Bartel 2009; Liu et al. 2009). Numerous miRs have been discovered to be involved in regulating the development of neuronal cells in mammalian brain and spinal cord (Bak et al. 2008; Kosik 2006). In addition to this some miRs have also been identified to play potential role in development of neurons and are believed to be important mediators contributing in cell differentiation (Kosik 2006). Studies earlier have showed that SCI may cause aberrant expression of miRs, which is feature of many responses of secondary injuries such as apoptosis, inflammation and oxidative stress (Liu et al. 2010; Wang et al. 2017). A study recently has suggested that miRs could regulate apoptosis via mitochondrial apoptosis cascade in various disorders (Yune et al. 2007).

In the present work, we created an SCI rat model followed by microarray analysis for determining the expression profiles of miRs in the injured spinal cord tissue. In addition to this, the role of miR-466c-3p in spinal injury mediated apoptosis was evaluated followed by the mechanism involved in the mitochondrial apoptosis cascade was investigated.

## Materials and methods

### Cell culture

For the present work we selected murine N9 microglia cell line which was procured from the Institute of Neuroscience, IVth Military Medical University, China. The use of cell lines received approval from the ethical board of IVth Military Medical University, China. The cell lines were cultured and stored in Dulbecco’s modified Eagle’s medium/F12 (DMEM) media supplemented with fetal bovine serum (10%) (FBS), streptomycin and penicillin (100 U/ml) maintained at 37 °C under humid conditions with 5% CO_2_ in an incubator.

### Cell treatments

The selected N9 microglia cell lines were exposed to various concentrations of hydrogen peroxide (30% w/w) (Sigma-Aldrich USA) for 10 h for inducing cell injury. The concentrations of hydrogen peroxide were 50 to 400 μM in phosphate buffered saline solution.

### Experimental animals and spinal cord injury model

For creating SCI animal model, we selected adult Sprague–Dawley rats (female) aging about 6 weeks and weighing between 210 and 240 g. The animals were obtained from the Beijing Jishuitan Hospital, Beijin, China. The animal study protocols were approved by animal ethical review board of Beijing Jishuitan Hospital, Beijin, China. The rats were housed in polypropylene cages under pathogen free conditions with relative humidity ranging between 55 and 65% and temperature 23 ± 1 °C following 12 h dark–light cycle. The rats were given free access to water and food (pellet diet). The rats were distributed into four groups viz. the SCI group (control), Sham operated group, miR-466c-3p mimics and antago-miR-466c-3p group (NC control), and all the groups had 8 rats. For inducing SCI, the rats were subjected to weight drop injury to the spinal injury at the T10 spinal disc using an impactor. For sham operated group the rats were subjected to T10 laminectomy without performing the weight drop injury. In the miR-466c-3p mimics treated group the rats were subjected to SCI and the received intrathecal dose of 1 μl/h miR-466c-3p mimics (20 nmol/ml) for next 3 days. For the negative control (NC) rats the SCI induced rats were given treatment of miR-466c-3p antagomir via intrathecal route (1 μl/h, 20 nmol/ml).

The rat model of spinal cord injury was created by submitting the rats to pentobarbital anesthesia followed by laminectomy of the T9–T10 level for revealing the spinal cord tissue, care was taken that no damage was done to the dura. The spine was stabilized by clamping the T8 and T11 processes, after this the dorsal surface of spine was submitted to weight drop injury using an impactor as reported earlier (Basso et al. 1995). The sham operated rats were submitted to laminectomy without performing the weight drop process for injury.

### miRNA microarray analysis

The spinal tissue of SCI injured rats were assessed for miRNA expression by miR micro array analysis, at least 2 rats from each group were subjected to Pentobarbital anesthesia and sacrificed after 2 weeks of SCI. The rats were operated and a 10 mm piece of spinal cord which also included the epicenter of injury was dissected and freezed in liquid nitrogen. The spinal tissue was processed with TRIzol reagent and total RNA was isolated and purified with the help of RNeasy kit (Qiagen, Germeny) following the supplied instructions. The samples were analyzed for RNA content using spectrophotometer (ThermoFisher USA), the isolated miRs were then hybridized on a LNA array. The resultant slides were scanned using an Axon Genepix microarray scanner. The images were analyzed using GenePix software version 6. The miRs showing intensities greater than or equal to 50 in all the samples were employed for calculating the normalization factor. The miRs were studied by the volcano plot filtering method.

### Isolation of RNA and qRT-PCR

Isolation of RNA was done from the spinal cord tissue isolated from the SCI rats, care was taken that the tissue had the damaged portion. TRIzol reagent (ThemoFisher USA) was used for isolating RNA following the supplied instructions. The reverse transcription was done using the Taqman RT kit (ThermoFisher USA) on a Real Time PCR system (ThermoFisher USA). The conditions maintained for thermocycling were temperature of 50 °C for first 2 min and then temperature of 95 °C for next 10 min, and then forty cycles of 15 s at 95 °C and next 10 min at 60 °C. The primer sequences which were utilized for the study were: miR-466c-3p, 3′-CACACAUACACACGUACAUAU -5′ (Forward) and 5′-AUCAGCCGGACCGCGCACCTT CACGC-3′ (Reverse); Bcl-2, 5′–CUAUGCAUCUUGUAACAUGUAUU–3′ (forward) and 5′–GGTGAGGACTCCAGCCACAA–3′ (reverse), all reactions were performed in triplicate.

### Behavioral parameters study

For assessing the behavioral parameters Basso, Beattie and Bresnahan (BBB) scoring was selected for evaluating the locomotor behavior in rats. The study was performed for an interval of 4 min by two trained and experienced investigators also they were kept blank about the experimental conditions. Open field locomotor test was performed for evaluating the hind limb function and the score was calculated in accordance to BBB scale as published earlier (Basso et al. 1995; Tang et al. 2014).

### Evaluation of lesion volume

For assessing the volume of lesion after SCI or after treating the rats with miR-466c-3p mimics, the rats were subjected to Sodium pentobarbital anesthesia (50 mg kg) via intraperitoneal route. The rats were submitted to trans-cardiac perfusion with isotonic saline solution (0.9%) followed by paraformaldehyde solution (4%) in 0.1 M Phosphate buffered saline pH 7.4 for 30 min. About 10 mm portion of spinal cord bearing injured part was harvested and then fixed in paraformaldehyde solution at 4 °C. For obtaining the sections the tissues were fixed in paraffin to form blocks. The sections were obtained using a rotary microtome of 10 μm thickness; the sections primarily covered the lesion sites which were then fixed on slides. The sliced tissue sections were stained with Cresyl violet acetate solution (0.5%) for 1 h and the slides were then imaged using Olympus microscope, the damaged area in the sections were quantified with the help of Image-pro software version 6 (Media Cybernetics, USA). The left over portion showing normal tissue area with preserved structure was considered as spared tissue and was reported. The section of tissue with least spared tissue percentage was regarded as the injury epicenter. The range of lesion epicenter was about 400 μm caudal and rostral, the area was evaluated through a distance of about 1800 μm in the periphery of injured epicenter for the total spared tissue percentage.

### TUNEL assay

For detecting apoptosis, the spinal cord tissue sections previously generated were submitted for TUNEL staining using a TUNEL apoptosis kit (ThermoFisher USA). The tissue sections were dipped into TUNEL reagent for 60 min, after this the nuclei were stained with DAPI solution (1 μg/ml) for 10 min and then the sections were immersed in Fluoromount media (SigmaAldrich USA). The positive cells were quantified in 10 random field in each slide using florescent microscope (Olympus), the apoptotic cells as well as total cells were counted.

### Luciferase reporter assay

Bioinformatics analysis was performed using TragetScan (http://www.targetscan.org/vert_72/) for identifying the favorable binding site between miR-466c-3p and Bcl-2. For the transfection, the miR-466c-3p mimics and inhibitor were used. The 3‘UTR fragment of Bcl-2 wild type as well as mutant were amplified and then cloned in reporter luciferase vector. Further, site directed mutagenase of 3′UTR of Bcl-2 was done on the identified binding site of miR-466c-3p with the help of site directed mutagenesis kit. Subsequently, N9 microglia cells were seeded at density of 2 × 10^5^ cells/well in the 24 well plates followed by co-transfection with p-miR-Bcl-2 3′UTR or p-miR-Bcl-2-mutant-3′UTR and miR-466c-3p mimics/inhibitor or negative control with the help of transfecting reagent Lipofectamine (ThermoFisher USA). Renilla luciferase was selected as control for normalizing the cell number after 48 h of transfection. The luciferase assay was performed with the help of luminescent reporter gene assay (ThermoFisher USA).

### Western blot analysis

The spinal cord segments having the injured site were harvested and were processed to isolate proteins, the proteins were also isolated from the N9 microglia cells with the help of radio immuno-precipitation buffer (Roche) as discussed in earlier studies (Wang et al. 2017). The resultants’ were mixed and centrifuged at 12,000 rpm for 15 min. The supernatants were removed and stored freezed. The expression of proteins was estimated from supernatant using bicinchoninic acid assay also known as BCA assay. About 30 μg protein was submitted for electrophoresis on SDS/PAGE (10%) and then transferred to PVDF membrane. The membranes were blocked with the help of non-fat milk (5%) for 12 h at 4 °C and then incubated with I^ry^ antibodies against Bax (1:1000), Bcl-2 (1:1000), Pro-caspase-3 and cleaved caspase-3 (1:1000), Pro-caspase-9 and cleaved caspase-9 (1:1000) and lastly the loading control Actin (1:1000). All the antibodies were bought from Santa Cruz Biotech. USA. The membranes were incubated for 60 min with anti-mouse IgG horse-radish peroxidase–conjugated secondary antibody. The bands were studied with the help of ImageQuant version 5.2 software.

### Immunohistochemical staining

For removing the blood the spinal tissue was submitted to intracardial perfusion with isotonic solution (0.9% sodium chloride) followed by phosphate buffered saline solution of paraformaldehyde solution (4%) for 30 min. The spinal cord section measuring 10 mm having the injured site was isolated and fixed in paraffin block and sections were produced of thickness 5 μm. The sections were deparaffinized using xylene followed by rehydrating them with series of alcoholic dilutions ranging from 100 to 40%. After this the sections were submitted to microwave in 10 mM citrate buffer for 5 min for epitope unmasking. After this the endogenous peroxidase was inactivated by treating the sections with H_2_O_2_ (3%) for 10 min. The sections were rinsed with phosphate buffered saline three times and were obstructed with fetal bovine serum (10%) and then incubated with anti-cleaved-caspase-3 antibodies (1:1000). The tissue sections were then again incubated along with anti-mouse IgG horseradish peroxidase IIry antibodies for 30 min. At last the sections were evaluated for immunoreactivity by 3,3′–diaminobenzidine staining for 3 min and then were cover sliped. The images were recorded using Olympus light microscope and quantification was preformed with the help of Aperio software version 9.

### Flow cytometry for apoptosis studies

About 1 × 10^6^ N9 microglia cells were collected and rinsed in phosphate buffered saline for 30 min. Fluorescein isothiocyanate (FITC) and Annexin V staining was done to identify the extent of apoptosis. The cells were rinsed at least thrice with phosphate buffered saline followed by incubation with propidium iodide (PI) (1 μl) and Annexin V–FITC (5 μl). After staining the cells were analyzed using flow cytometry (ThermoFisher USA).

### Determination of Caspase-3 activity

Colorimetric assay was done for determining caspase-3 activity using a kit following the supplied instructions (SigmaAldrich USA). The N9 cells were isolated by centrifugation at 12,000 rpm for 10 min and then incubated in lysis buffer for 15 min. Further, the lysates were centrifuged at 12,000 rpm for 15 min, later the protein content was determined by protein estimation kit (SigmaAldrich USA) following the supplied instructions. The isolated lysates were incubated with Ac-DEVD-pNA (0.2 mM) (10 μl) reaction buffer for 2 h. The samples were evaluated using microplate reader at 405 nm.

### Statistical evaluation

The statistical studies were performed using Graphpad Software version 6. The results were presented as mean ± SD. Spearman’s rank correlation was done establishing the link between expression of miR-466c-3p and Bcl-2. The variations in the groups were evaluated by one-way ANOVA tukey’s post hoc test and the variations in the group were analyzed by student’s t-test. P < 0.05 was considered as significant.

## Results

### SCI causes aberrant expression of miRNA in rats

**Fig. 1:**
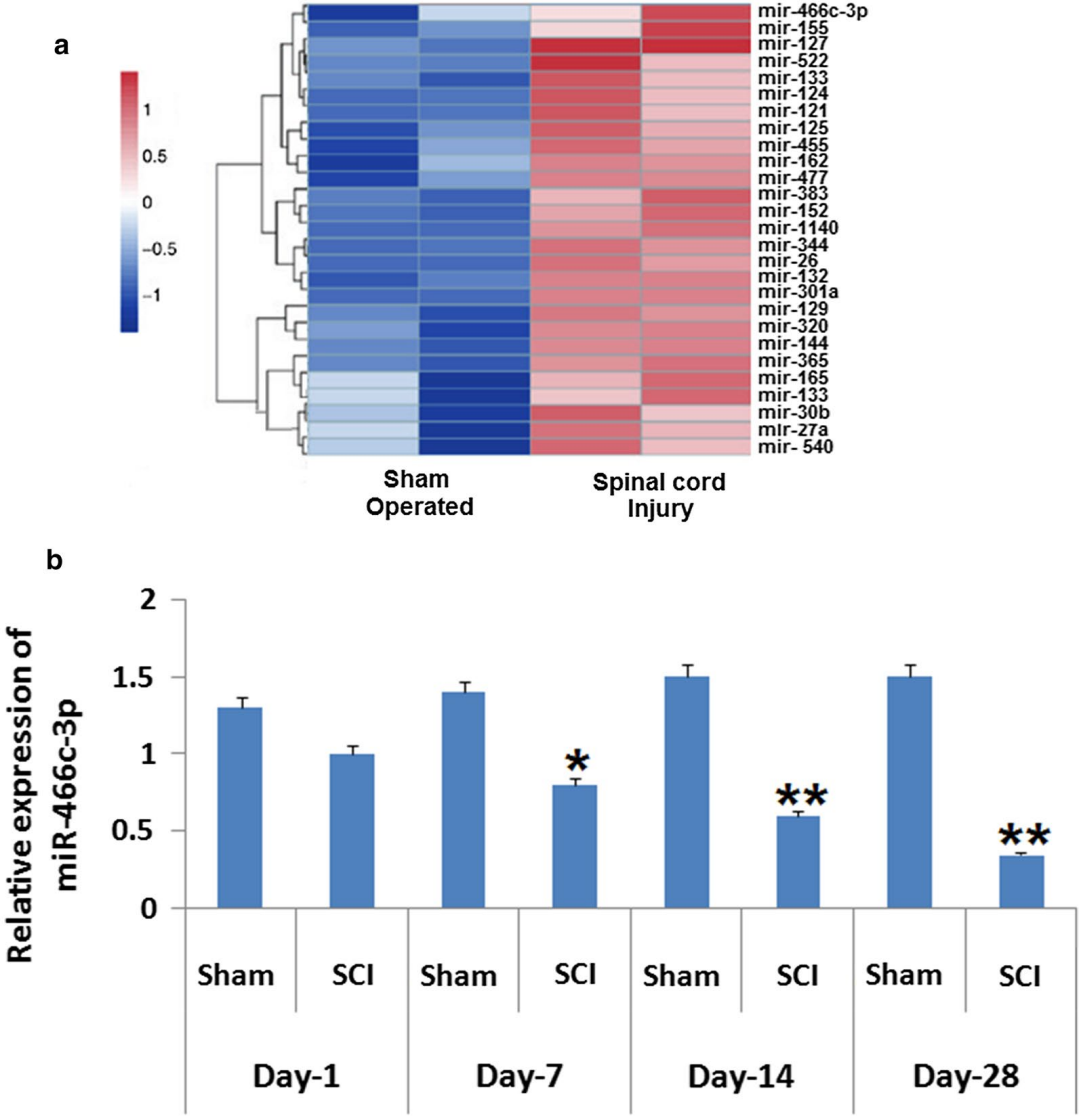
Expression of miR in rats subjected to spinal cord injury. **a** Outcomes of heat map analysis showing significant changes in expression of miRs in rats after 14 days subjecting them to spinal cord injury. The blue color shows suppression and red shows over-expression. **b** Quantitative results of reverse transcription by qRT-PCR for determining the expression of miR-466c-3p in the spinal cord tissues of rats isolated on the 1st, 7th, 14th and the 28th day after inducing spinal cord injury. The results are presented mean ± SD. *P < 0.05, **P < 0.01 compared to sham operated animals

In line to evaluate the potential role of miRs in SCI, we created a rat model of SCI followed by microarray analysis for identifying the expression of miRs in the injured spinal cord. We found that expressions of large number of miRs were altered after 2 weeks inducing SCI and among them miR-466c-3p was the most significantly suppressed compared to the sham operated rats (Fig. 1a). In a previous research it was observed that the expression miR-466c-3p decreased significantly with time under spinal injured conditions in rats (Druz et al. 2011). Therefore, qRT-PCR analysis was performed for verifying the expression of miR-466c-3p in spinal cord tissues i.e. on 1st, 7th, 14th and 28th day after inducing spinal cord injury. The outcomes suggested a significant down-regulation in levels of miR-466c-3p in SCI group against the sham operated group observed from the 7th to the 28th day (P < 0.05; Fig. 1b). The findings indicated that SCI causes ectopic expression of miRs in spinal cord tissues and miR-466c-3p may hold the pathogenesis in animal model of SCI.

### Up-regulation of miR-466c-3p attenuated the functional activity and inhibited apoptosis of neuronal cells in SCI rats

**Fig. 2:**
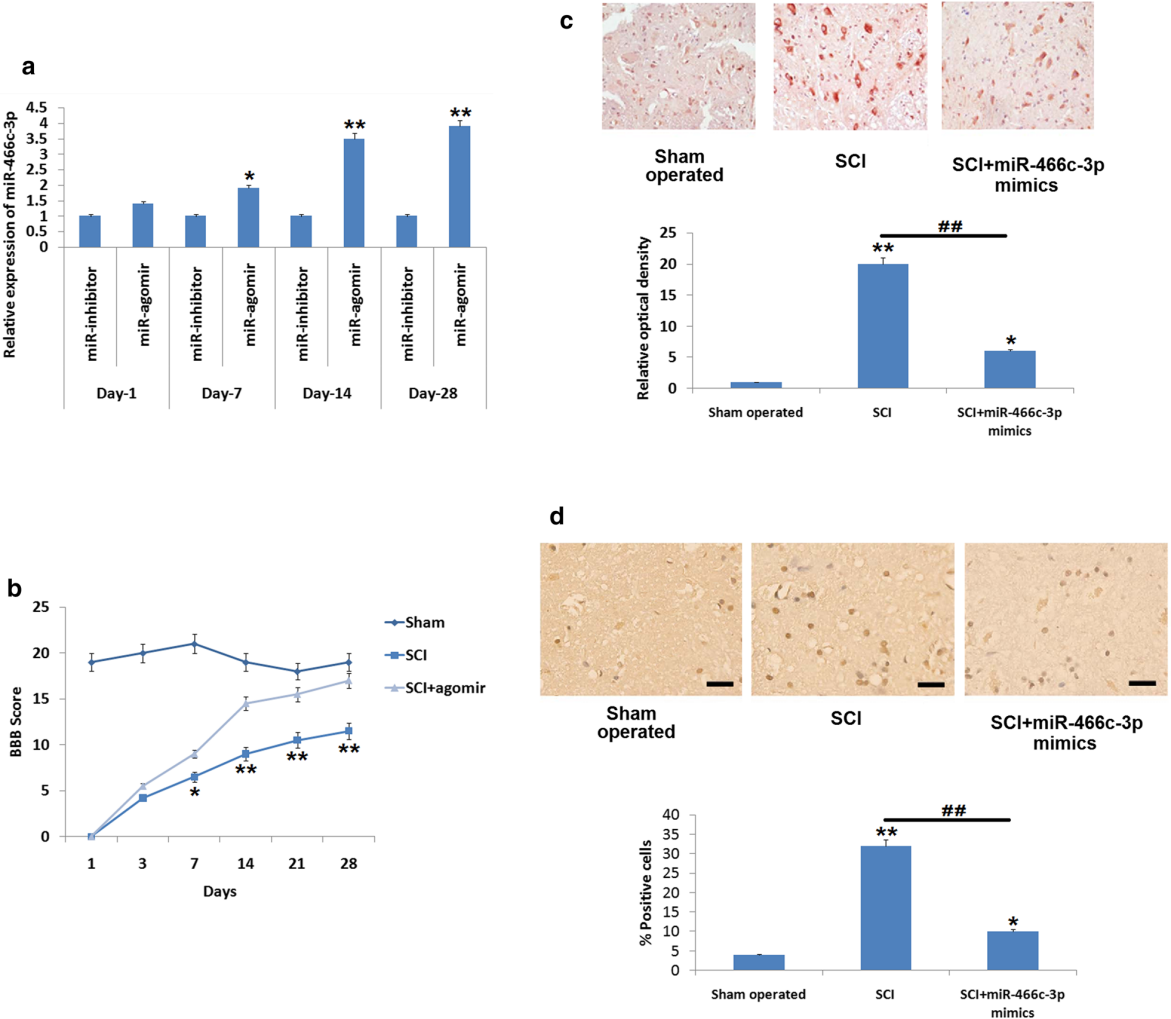
**a** Expression of miR-466c-3p was determined by qRT-PCR reaction on the 1st, 7th, 14th and the 28th day after inducing spinal cord injury followed by treatment of miR-466c-3p mimics. *P < 0.05, **P < 0.01 compared to respective miR-inhibitor negative control. **b** Outcomes of BBB analysis for evaluation of locomotor activity on the 1st, 7th, 14th and the 28th day after inducing spinal cord injury after treating the animals with miR-466c-3p agomir. **c** Results of Immunohistochemical analysis showing expression of cleaved caspase-3 in the spinal tissue after spinal cord injury. *P < 0.05, **P < 0.01 compared to sham operated animals; ##P < 0.01 compared to SCI rats. **d** Results of TUNEL assay for studying the apoptosis in the cells after 14 days of inducing spinal cord injury. The results are mean ± SD

To evaluate the function of miR-466c-3p in SCI induced rats, the SCI induced rats were treated with miR-466c-3p mimics given by intrathecal route. After this the spinal cord tissues were harvested and screened for over-expression of miR-466c-3p with the help of qRT-PCR. The results (Fig. 2a) showed that the relative expression of miR-466c-3p was significantly upregulated on the 7th as well as 28th day compared to the rats treated with miR-466c-3p inhibitor (i.e. agomiR-466c-3p NC) (P < 0.05), also it was found that the expression was highest at the 14th day.

In addition to qRT-PCR analysis for expression of miR-466c-3p, motor function was evaluated by Basso, Beattie, Bresnahan (BBB) scale in the SCI rats after treating them with miR-466c-3p mimics and agomiR-466c-3p NC, the outcomes suggested that upregulation of miR-466c-3p in the spinal cord injury rats treated with miR-466c-3p mimics showed significant improvement in motor function from the 7th day as compared to spinal cord injury rats (Fig. 2b, P < 0.05). The staining results of Cresyl violet suggested that the normal spared tissue area in sections of miR-466c-3p mimics treated SCI rats increased significantly compared to SCI induced rats, indicating that miR-466c-3p mimics decreases the volume of lesion in the spinal cord tissues after SCI. Further we evaluated using immuno histochemical staining whether miR-466c-3p could regulate the expression of apoptosis related protein i.e. cleaved caspase-3 in the spinal cord after SCI. As evidenced, SCI caused a substantial overexpression of cleaved–caspase–3 in the spinal tissues compared to sham operated rats (Fig. 2c), whereas, the treatment of miR-466c-3p mimics caused a significant decrease in levels of cleaved-caspase-3 (Fig. 2c, P < 0.01). In addition, TUNEL studies were done to assess apoptosis of neuronal cells. The number of TUNEL positive cells increased substantially in rats submitted to SCI against the sham operated rats (P < 0.01), however it was observed that miR-466c-3p mimic suppressed TUNEL positive cells in rats compared to SCI induced rats (P < 0.01 Fig. 2d). These findings indicated that miR-466c-3p mimic attenuated motor function, decreases the volume and size of lesion and decrease apoptosis of neuronal cells post SCI.

### miR–466c-3p decreases the expression of Bcl-2 via 3′–UTR in N9 microglia cells

**Fig. 3:**
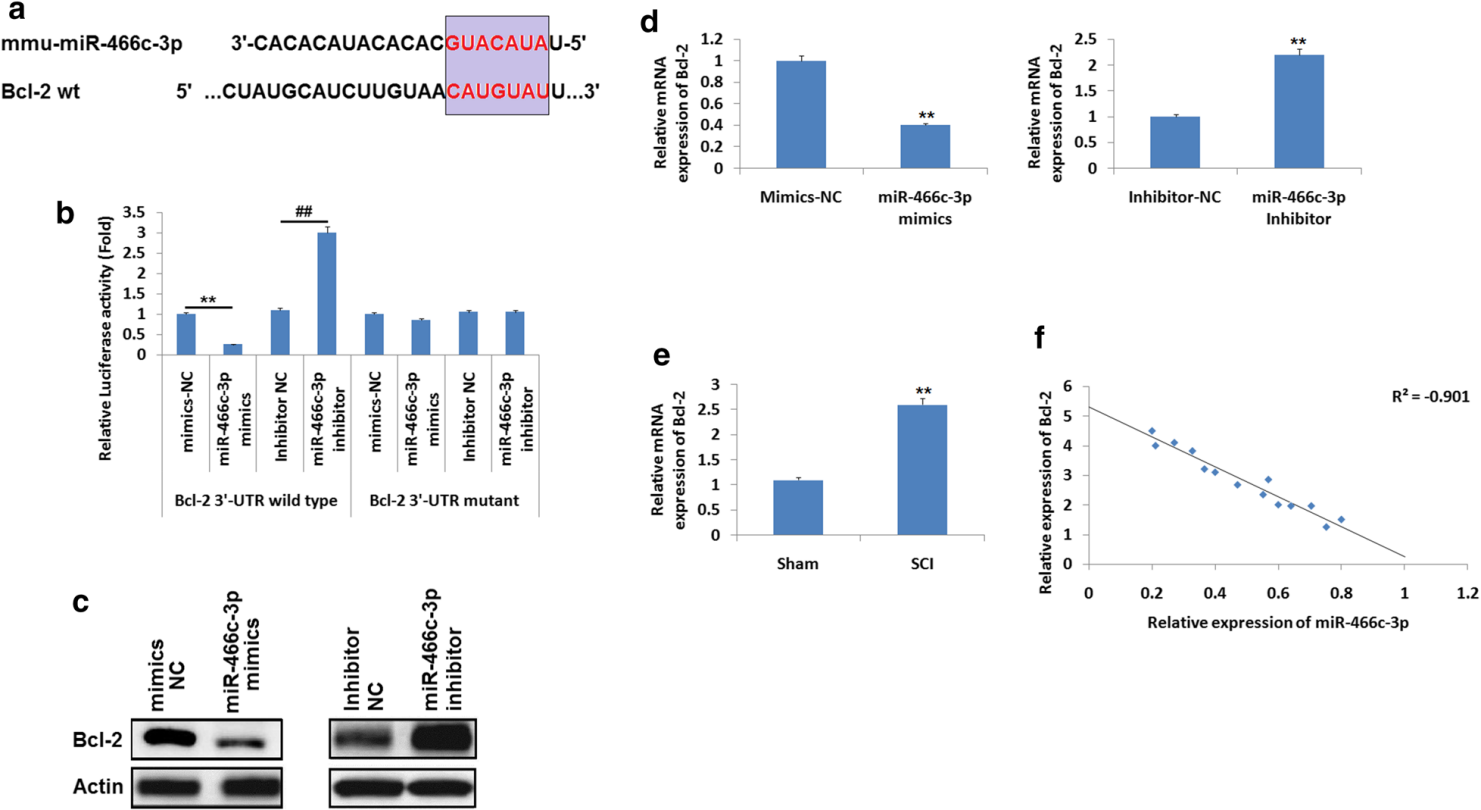
**a** Bcl-2 showed potential binding site for miR-466c-3p in the N9 microglia cells on the 3′UTR region of Bcl-2 as predicted by TargetScan. **b** Resulst showing relative luciferase activity of Bcl-2 wild type or mutant 3′UTR of N9 microglia cells after transfection them with miR-466c-3p mimics or inhibitor or negative control. **P < 0.01 compared to mimics negative control; ^##^P < 0.01 compared to inhibitor negative control. **c** Western blot analysis for expression of protein levels of Bcl-2. **d** mRNA levels of Bcl-2 determined by qRT-PCR, actin was selected as loading standard. **P < 0.01 compared to negative control. **e** Results of qRT-PCR analysis for determining the mRNA levels of Bcl-2 in the injured spinal cord tissue after 14 days of injury. **f** The results suggested a inverse correlation between expression of miR-466c-3p and Bcl-2 in the spinal tissues post 14 days of injury (R^2^ = − 0.901). The results are mean ± SD

Studies earlier have confirmed that miR-466 shows protective effect against apoptosis in mammalian cells (Rojo et al. 2014). Hence it was hypothesized that miR-466c-3p may affect apoptosis of neurons in rats subjected to SCI via altering Bcl-2. For predicting potential targets of miR-466c-3p, bioinformatics analysis was performed, it was evidenced that Bcl-2 was target gene of miR-466c-3p having potential site located on the 3′–UTR region (Fig. 3a). To corroborate the in silico predicted target, the wild type (WT) and mutant (mut) type Bcl-2 3′-UTR was prepared and was put into the firefly p-miR reporter luciferase vector. The pathological factors were studied in N9 microglia cells. The N9 cells were transfected with either miR-466c-3p mimics or inhibitor NC followed by evaluation of luciferase activity. The results suggested that, as compared to inhibitor NC the miR-466c-3p mimics caused significant inhibition of luciferase activity along with wild type 3′UTR, whereas miR-466c-3p inhibitor increased the luciferase activity significantly compared to inhibitor (Fig. 3b, P < 0.01). In addition to this, miR-466c-3p do not suppressed the luciferase activity in the 3′UTR of Bcl-2 containing reporter vector having mutations in the miR-466c-3p binding site (Fig. 3b). To confirm whether, expression of Bcl-2 is negatively regulated by miR-466c-3p, both qRT-PCR and western blot analysis was performed for detecting the mRNA as well as protein levels of Bcl-2. The results demonstrated that, upregulation of miR-466c-3p suppressed the expression of Bcl-2 at both the protein as well as at mRNA levels in N9 microglia cell lines (Fig. 3c, d). Inversely, suppression of miR-466c-3p enhanced the expression of Bcl-2 at mRNA as well as at protein levels (Fig. 3c, d). The outcomes of qRT-PCR were used to evaluate the mRNA levels of Bcl-2 in the spinal cord tissues (n = 8). We found that the mRNA levels of Bcl-2 increased significantly in SCI rats compared to sham operated (Fig. 3e, P < 0.01). Statistical analysis suggested a significantly negative correlation between expression of miR-466c-3p and Bcl-2 in the spinal cord tissues of SCI rats (r = − 0.901; Fig. 3f). Altogether, the outcomes suggested that miR-466c-3p suppressed the levels of Bcl-2 via targeting the 3′UTR of N9 cells directly, indicating Bcl-2 may be the potential target of miR-466c-3p in the injured spinal cord.

### Upregulation of Bcl-2 halts the attenuating effect of miR–466c-3p on hydrogen peroxide exposed N9 microglia cells

**Fig. 4:**
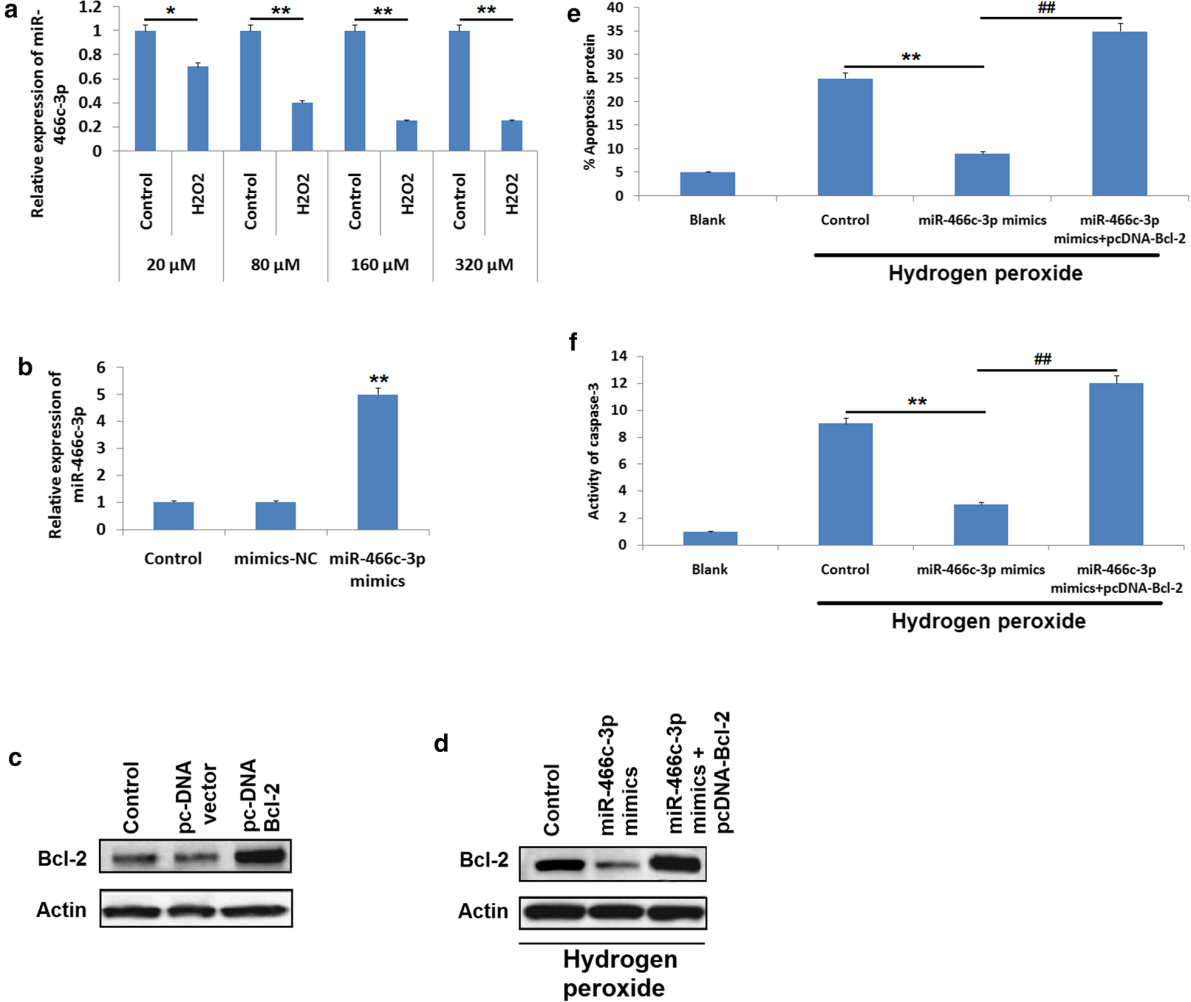
**a** The N9 microglia cells were treated with hydrogen peroxide of varied concnetrions for 10 h followed by measuring the expression by qRT-PCR. *P < 0.05, **P < 0.01 compared to control. **b** qRT-PCR analysis for expression of miR-466c-3p in N9 microglia cells transfected with miR-466c-3p mimics or negative control.*P < 0.05, **P < 0.01 compared to mimice neative control group cells. **c** The N9 cells received transfection of pc-DNA-Bcl-2 or pc-DNA-vector followed by evaluation of Bcl-2 by western blot analysis. **d** The N9 cells were transfected with miR-466c-3p mimics or miR-466c-3p mimics along with pc-DNA-Bax and expression of Bcl-2 were estimated by western blot analysis. The cells were treated with hydrogen peroxide followed by transfection with miR-466c-3p mimics or miR-466c-3p mimics and pc-DNA-Bcl-2. **e** The number of cells under gone apoptosis and **f** activity of caspase-3 were evaluated with the help of flow cytometry and colorimetric analysis respectively. **P < 0.01, ##P < 0.01 compared to control and mimics respectively

Previous reports have indicated that reactive oxygen species (ROS) play an important role in SCI as ROS can trigger numerous cascade of apoptosis, hydrogen peroxide exposed N9 cells have been used as cellular models of SCI for examining the pathological factors after SCI (He et al. 2000). In the current study, N9 microglia cells were submitted to H_2_O_2_ at varied concentrations 20–320 μM for 10 h and levels of miR-466c-3p were evaluated with the help of qRT-PCR analysis (Fig. 4a). It was found that exposure of H_2_O_2_ to N9 cells caused a significant suppression of miR-466c-3p, also the suppression was dose mediated (P < 0.05). In addition to this, qRT-PCR and immunoblot study was done to assess the upregulation efficacy of miR-466c-3p or Bcl-2. The outcomes suggested that, expression of miR-466c-3p and Bcl-2 was upregulated in N9 cells exposed to pc-DNA-BAx and miR-466c-3p mimics respectively (Fig. 4b, c). Also, it was noticed that up-regulation of miR-466c-3p suppressed the Bcl-2 protein levels in H_2_O_2_ exposed N9 cells (Fig. 4d). The present outcomes suggested that, up-regulation of miR-466c-3p caused a significant decrease in apoptotic cells among the hydrogen peroxide treated N9 cells compared to control, however it was observed that the attenuating effect of miR-466c-3p on N9 cells was blocked significantly with upregulation of Bcl-2 (Fig. 4e, P < 0.01). Also, over-expression of Bcl-2 halted the effects of miR-466c-3p blockade activity of caspase-3 in hydrogen peroxide exposed N9 cells (Fig. 4f, P < 0.01). All together, the findings indicated that miR-466c-3p could suppress apoptosis in neuronal cells via suppressing Bcl-2 in hydrogen peroxide exposed N9 microglia cells.

### miR-466c-3p over-expression inhibits the mitochondrial apoptotic pathway

**Fig. 5:**
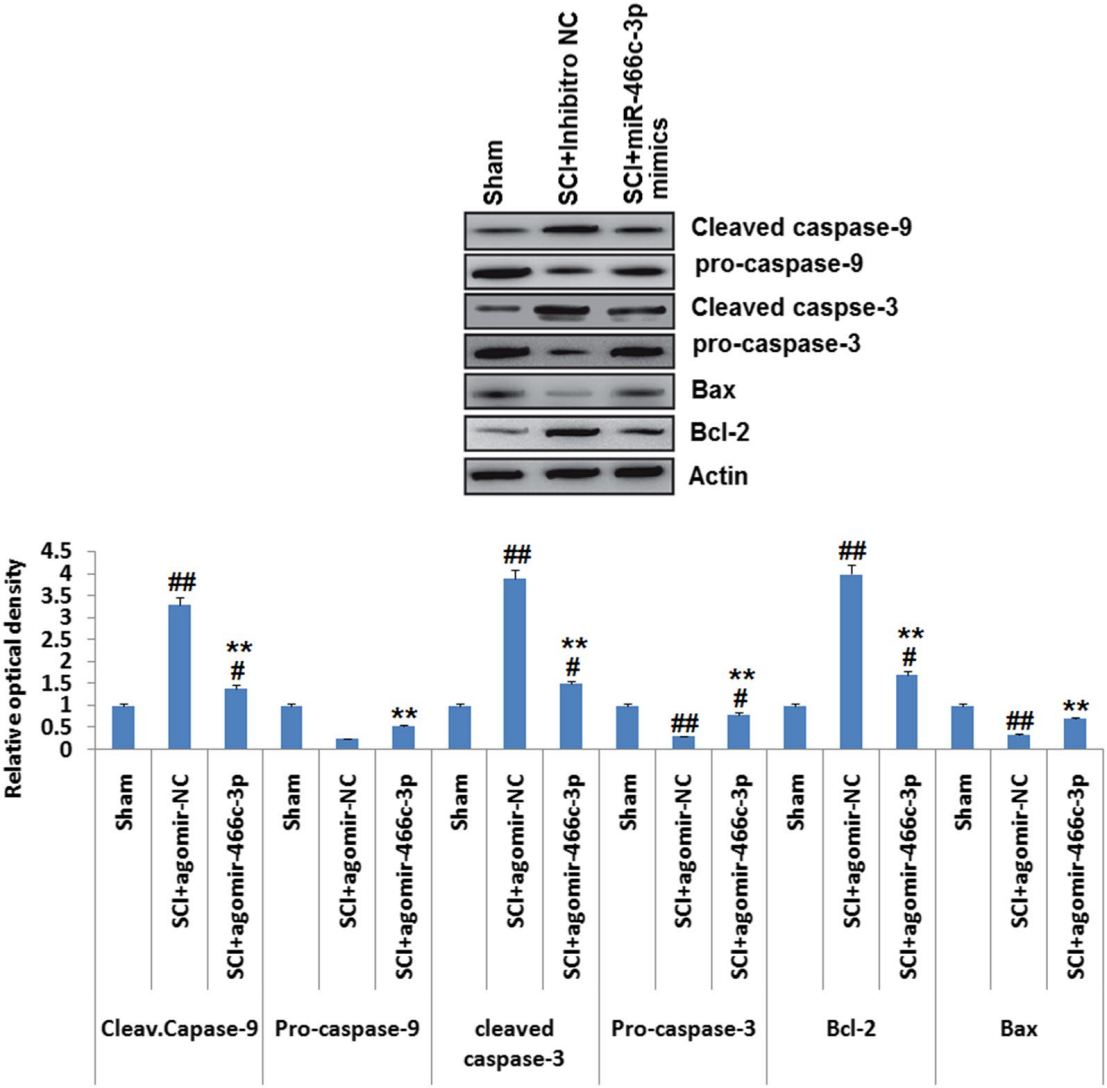
The experimental animals were induced with spinal cord injury and were exposed to miR-466c-3p mimics or agomir-negative control followed by western blot analysis for determining the expression of cleaved-caspse-9, cleaved-caspase-3, pro-cspase-3, Bcl-2 and Bax in the spinal cord tissue subjected to injury, actin was selected as loading control. The results are mean ± SD. **P < 0.01 compared to spinal cord injury rats + treated with agomir-negative control; #P < 0.05, ##P < 0.01 compared to sham operated rats

The pro-apoptotic proteins Bax and Bcl-2 can cause liberation of cytochrome C (Liu et al. 2008; Noguchi et al. 1999). In apoptosis process, overexpression of Bcl-2 can enhance the process by blocking Bax (Klapsinou et al. 2015). Death receptor apoptosis and mitochondrial apoptosis are the two important apoptosis pathways (Su et al. 2016; Lu et al. 2017a, b). Bcl-2 is identified as one of the important molecule responsible for regulating mitochondrial apoptosis pathway which promotes the release of cytochrome c in the cytoplasm from mitochondria (De Biase et al. 2005). In line to evaluate, whether miR-466c-3p is involved in regulating the mitochondrial apoptosis pathway via down- regulating the expression of apoptosis associated proteins in SCI rats, western blot study was performed for assessing the expression of Bcl-2, Bax, Caspase-3/-9 and Cleaved Caspase-3/-9 in spinal tissues of rats. We evidenced that the levels of Cleaved Caspase -3/-9 and Bcl-2 were significantly increased whereas, expression of Caspase-3/-9 and Bax were suppressed significantly in SCI rats treated with NC inhibitor compared to sham operated rats. However, upregulation of miR-466c-3p caused a significant decrease in levels of cleaved caspase -3/-9 and Bcl-2 also caused a significant increase in levels of Caspase-3/-9 and Bax in SCI rats treated with miR-466c-3p mimics compared to NC inhibitor rats (Fig. 5, P < 0.01). These findings suggested that, upregulation of miR-466c-3p may inhibit the mitochondrial apoptotic pathway via suppression of Bcl-2 in rats after SCI.

## Discussion

Spinal cord injury leads to alterations at both molecular as well as at biochemical levels, these changes lead to generation of free radicals, release of inflammatory mediators, death of neurons and axonal plasticity (Hu et al. 2013). Earlier, some studies have suggested that spinal cord injury may lead to abnormal expression of miRs and these miRs may alter the functional outcomes as well as the pathophysiology (Malhotra et al. 2001). However, studies highlighting the functional significance of some specific miRs are lacking. Here, we constructed an animal model of SCI followed by microarray analysis for expression of miRs at various time points after inducing SCI. We observed that SCI caused deregulation in expression of miRs in rats submitted to SCI among the miRs, miR-466c-3p was the most significantly down-regulated in the spinal cord tissue. Also, it was found that upregulation of miR-466c-3p attenuated SCI by improving functional recovery, decrease in the size and volume of lesions and also deceased apoptosis. It was also evidenced that, upregulated miR-466c-3p caused inhibition of Bcl-2 in N9 microglia cells via binding to the 3′UTR region, this was confirmed when it was tested that upregulated Bcl-2 caused inhibition in the protective effect of miR-466c-3p in the N9 cells exposed to hydrogen peroxide. The present work demonstrated the protective role of miR-466c-3p in SCI animal model via blocking the mitochondrial apoptosis cascade.

Literature previously has confirmed that miR-466c-3p is one of the aberrantly expressed miR in the neurons (Druz et al. 2011). The study showed that miR-466c-3p was one of the most down-regulated miR during the SCI specifically during the 13th and 14th day post-SCI. Parallel to these findings, the present results showed that SCI caused aberrant expression miRs and miR-466c-3p was one of the most significantly suppressed miR in the rats subjected to SCI. The present study also suggested that, up-regulation of miR-466c-3p in rats by treating them with miR-466c-3p mimics improved the motor function as evaluated by BBB scoring; decreased the lesion volume and also suppressed the apoptosis of neuronal cells in SCI induced rats. These findings demonstrated that miR-466c-3p shows protective role on rats induced to SCI.

Mitochondria play a vital role in the process of apoptosis by releasing important mediators such as cytochrome c (Zou et al. 1999). Bcl-2 is an important apoptosis regulator, it has been identified to increase the permeability of the mitochondrial membrane causing release of cytochrome c in the cytoplasm (Zou et al. 1999). Cytochrome c is responsible for amplifying the caspase-9 pathway in the mitochondrial apoptosis cascade which in response triggers Caspase-3 to active Caspase-3 (Zou et al. 1999). In the present study, miR-466c-3p suppressed the expression of Bcl-2 via targeting 3′UTR in N9 microglia cells. In addition to this, the results of correlation analysis suggested a negative correlation between expression of miR-466c-3p and Bcl-2 in spinal cord tissue of SCI induced rats, indicating Bcl-2 as a potential target of miR-466c-3p in rats. Hence, it may be speculated that miR-466c-3p may modulate the mitochondrial apoptosis cascade via blocking the expression of Bcl-2 in SCI rats. The present findings suggested that over-expression of miR-466c-3p suppressed the expression of Bcl-2, cleaved caspase-3/-9 and enhanced the expression of Bax, Pro-caspase-3/-9 in the spinal cord tissues of SCI rats. Altogether, the results suggested that miR-466c-3p may exhibit protective effect in SCI via inhibiting the mitochondrial apoptosis pathway. However, further studies are needed for evaluating in-depth molecular mechanism for miR-466c-3p in SCI.

The current work showed that SCI causes abnormal expression of miR in SCI induced rats, among them miR-466c-3p was one of the highly suppressed miRNAs in spinal cords of SCI rats. Also it was noticed that overexpression of miR-466c-3p improved the motor function, decreased lesion size and volume and suppressed apoptosis of neurons in rats subjected to SCI. The present findings also suggested that miR–466c-3p targets Bcl-2 in N9 microglia cells and can extend attenuating effect in SCI via inhibiting the apoptosis cascade in mitochondria, proposing that miR–466c-3p may be a potential and novel therapeutic target in treating SCI.

## Data Availability

The supporting data for present findings are under ethics laws of university and is hence not presented here.
